# A guide to best practice in faculty development for health professions schools: a qualitative analysis

**DOI:** 10.1186/s12909-022-03208-x

**Published:** 2022-03-05

**Authors:** Samar A. Ahmed, Nagwa N. Hegazy, Archana Prabu Kumar, Enjy Abouzeid, Nourhan F. Wasfy, Komal Atta, Doaa Wael, Hossam Hamdy

**Affiliations:** 1 Forensic Medicine Ain Shams University, Cairo, Egypt; 2 Faculty of Medicine, Menoufia University (MU), Menoufia, Egypt; 3 Medical Education Unit, College of Medicine and Medical Sciences, Arabian Gulf University, Manama, Bahrain; 4 Department of Physiology, Sri Ramachandra Medical College and Research Institute, SRIHER, Chennai, Tamil Nadu India; 5 Medical Education Department, Faculty of Medicine, Suez Canal University, Ismailia, Egypt; 6 The University of Faisalabad Pakistan, Faisalabad, Pakistan; 7 Pediatric Surgery & Medical Education and Chancellor, Gulf Medical University, Ajman, United Arab Emirates

**Keywords:** Faculty development, Evaluation, Indicator

## Abstract

**Background:**

This is a practice guide for the evaluation tool specifically created to objectively evaluate longitudinal faculty development programs (FDP) using the “5×2 -D backward planning faculty development model”. It was necessary to create this tool as existing evaluation methods are designed to evaluate linear faculty development models with a specific endpoint. This backward planning approach is a cyclical model without an endpoint, consisting of 5 dynamic steps that are flexible and interchangeable, therefore can be a base for an evaluation tool that is objective and takes into account all the domains of the FDP in contrast to the existing, traditional, linear evaluation tools which focus on individual aspects of the program. The developed tool will target evaluation of longitudinal faculty development programs regardless of how they were planned.

**Methodology:**

Deductive qualitative grounded theory approach was used. Evaluation questions were generated and tailored based on the 5 × 2-D model followed by 2 Delphi rounds to finalize them. Based on the finalized evaluation questions from the results of the Delphi rounds, two online focus group discussions (FGDs) were conducted to deduce the indicators, data sources and data collection method.

**Results:**

Based on the suggested additions, the authors added 1 new question to domains B, with a total of 42 modifications, such as wording changes or discarding or merging questions. Some domains received no comments, therefore, were not included in round 2. For each evaluation question, authors generated indicators, data sources and data collection methods during the FGD.

**Conclusion:**

The methodology used to develop this tool takes into account expert opinions. Comprehensiveness of this tool makes it an ideal evaluation tool during self-evaluation or external quality assurance for longitudinal FDP. After its validation and testing, this practice guide can be used worldwide, along with the provided indicators which can be quantified and used to suit the local context.

**Supplementary Information:**

The online version contains supplementary material available at 10.1186/s12909-022-03208-x.

## Introduction

Faculty Development Programs (FDPs) in Health Professions Education (HPE) encompass an array of programs and activities that are designed to enhance the expertise of educators in various domains including, but not limited to, teaching, assessment, educational research, curriculum design, mentorship, leadership, and accreditation [1, 2].

Steinert et al. [3] found that, for an FDP to be effective, it should be based on experiential learning; effective feedback; peer-reviewed concepts; collaborative learning; useful interventions; successful models and diverse educational strategies.

Moreover, a FDP in health professions education (HPE) is a well-recognized tool to promote Continuous Professional Development (CPD). CPD is a wider paradigm, encompassing all the core elements of HPE, including knowledge, professionalism and skills such as medical, social, personal, leadership and managerial skills [4].

A necessary part of implementing FDPs is regular evaluation. The evaluation of the effectiveness of most FDPs is reported in the literature by quantitative questionnaires and self-reporting tools [5]. Other techniques for evaluation include hierarchical models like “Kirkpatrick” and other various qualitative methodologies such as interviews [6, 7]. Several studies report how individual components of the FDP are efficient but the literature is scarce for comprehensive evaluation for the whole FDP [8].

The World Federation of Medical Education recommends a set of global standards to monitor the design, development, implementation, and evaluation of CPD [4]. These standards comprise 9 areas namely, “Mission & outcomes, Educational Program, Assessment & Documentation, Individual Doctor, CPD Provision, Educational Resources, Evaluation, Organization and Continuous Renewal”. These are further divided into 32 sub-areas [4]. All the identified components have intricate elements and dynamic links of communication between them. These standards, not only enable the identification of strengths and weaknesses of the FDP but also foster quality enhancement.

However, it is advised by the World Federation for Medical Education that a regulatory body from each country or institution should examine the applicable standards accordingly and build a fitting version that suits the local context. Moreover, standards for CPD programs essentially focus on the processes and procedures of training rather than the core of the training. FDPs based on such robust models are deemed a solid prerequisite to provide effective training for health professionals including doctors and nurses [9].

FDPs need to be geared for the improvement of the whole institutional atmosphere, including student and faculty skills, growth, organizational development, leadership and change management capacities [10]. To accomplish all this, a linear approach may fall short as it focuses on a rigid model with specific initiation and termination dates with very limited room for iteration. Similarly, using a single method of evaluation is deemed as an insufficient technique to judge all aspects of a multi-faceted program such as a FDP [10]. Therefore, there is a dire need for outcome measures and a well-designed study to rigorously evaluate the FDPs, justifying the time and resources requested by departments and institutions.

Several models have been put forth for Faculty development (FD). O’Sullivan et al., [11], proposed the significance of the four fundamental components of FDP, namely: the facilitators, participants, context, and program along with their associated practices, while Dittmar and McCracken [12] put forth the META model (Mentoring, Engagement, Technology, and Assessment) converging on personalized mentoring, constant engagement, the amalgamation of technologies and systematic assessments. This was embraced by regular objective evaluations done by all the stakeholders involved in the educational process, including self, students, and peers [12]. Furthermore, Lancaster in 2014, recognized “centres, committees, and communities” as three core areas in his FD evaluation model [13].

Most of these programs were designed and structured keeping in mind specific criteria and objectives, primarily geared towards strengthening the teaching skills, leadership and learners’ satisfaction [7]. Despite that, such longitudinal FDPs were recommended by many authors for reaping long-lasting benefits in terms of institutional accreditation and better patient care [14–19].

In 2020, this trend of linear FDP approaches was taken notice of by Ahmed S A et al., who devised a model based on the “Backward Planning Approach”. This was in response for the need for a more inclusive model. This model reinforces the fact that FD should be considered as a series of cyclical processes, rather than a single endpoint with no future visitations or evaluations of the implemented changes [20].

By “cyclical” we imply a continuous methodology that will assess the program at different points of its progression and then revisit those areas to reinforce and reevaluate issues in the form of a “circle” this is different from traditional linear models of evaluation, for example, the Kirkpatrick model. The Kirkpatrick model addresses the evaluation of FDP in a linear ascending fashion with levels of evaluation. As opposed to this the “5x2 D Model”, consists of five dynamic steps “Decide, Define, Design, Direct, Dissect” which are flexible and interchangeable as part of a cycle [20]. What sets this model apart from the rest reported in the literature, is its flexibility and adaptability.

The 5X2 D-model envisions FDP as an ongoing rejuvenating process of continual renewal and refreshment of skills, performance indicators and competencies. It comprises flexible domains that are revisited continuously. This reiteration and the provision of interchangeability make this cycle a dynamic model for FDP [20].

With the development of the ‘5x2 D Model’, it was necessary to create an evaluation tool suitable for FDP that utilize this model. This is done offering the additional benefit of creating an evaluation tool that is both objective and inclusive of all the domains of the FDP as a whole rather than its individual aspects.

Evaluation of such a holistic longitudinal FDP model needs to be rooted in rigorous methodology and must ensure achievement of the internationally recognized quality standards. Therefore, the purpose of our study is to develop, and face validate an evaluation guide for Health professions schools to use for assessing the progress of the longitudinal FDPs based on the “5X2 D-model.”

## Methodology

The Authors followed a deductive qualitative grounded theory approach aiming at generating descriptors for the evaluation of FDPs. This work utilized a qualitative multistage approach starting with the generation of the evaluation questions, Delphi technique and an expert consensus session followed by focus groups discussions (FGD), as outlined below:

### Step 1: generation of evaluation questions

**Fig. 1:**
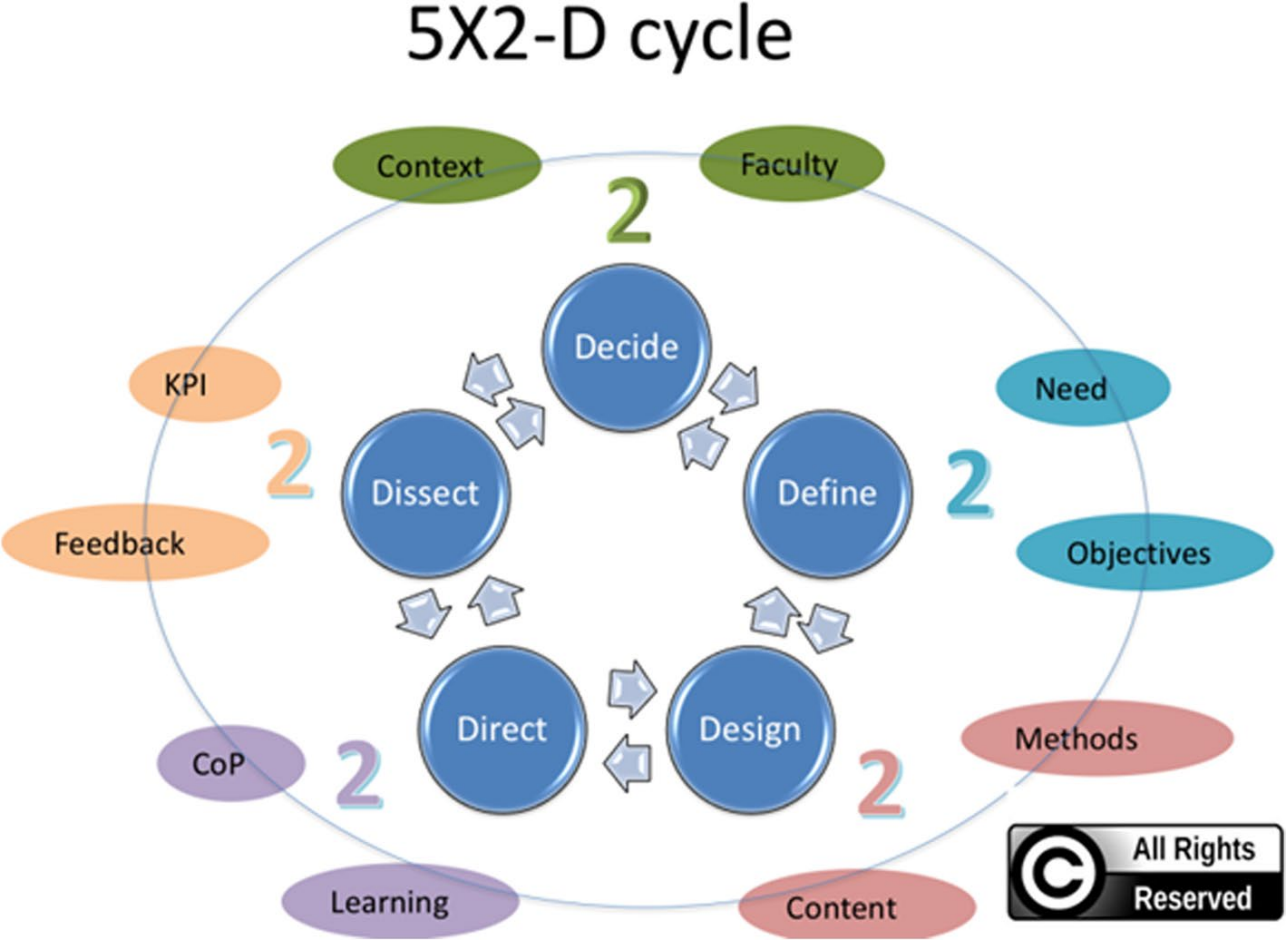
5X2 D cycle Backward Planning Model

Researchers generated the evaluation questions by reviewing the preceding similar appraisal work in the literature and adopting the 5 × 2 D Model (Fig. 1) [20] to analyze the data thematically to identify the proper evaluation questions for the FDP. This was done by the authors and the saturation was confirmed in a series of two virtual meetings, each lasting for 2 h.

### Step2: Delphi technique

To reach the consensus of the experts on the developed evaluation questions for the FDP, authors developed a survey and pilot-tested it on a group of five respondents.

Delphi approach was deployed over two- online rounds, conducted from May 2021 to June 2021. The Delphi panel consisted of 20 medical educators, purposefully chosen based on their experience in the domain of FD and managing quality standards. Nineteen educators participated in round one and eighteen educators participated in round two.

A consensus threshold of 100% was chosen as the cutoff for continuation, i.e., if 100% of the evaluation questions reached consensus by round 2, the study would be considered complete. This decision was based on a common observation of Delphi studies [21, 22].

### Consensus rules

Pre-determined consensus rules were used by the authors to guide decision-making regarding when the evaluation question was to be accepted or excluded. These rules were referenced in rounds 1 and 2. These rules were as follows:

- Consensus: Mean/average score is ≥4 on the 5-Point Likert Scale. Or percentage more than 75%.
- Non-consensus: Mean/average score is < 4 on the 5-Point Likert Scale.

The Experts were anonymous to each other throughout the study. The Delphi study was not completely anonymous as the authors are aware of experts’ identities. Each participant was assigned an alphanumeric identifier that was attached to their contributions.

Rounds 1 and 2 involved ranking the questions on a 5-point Likert scale. This allowed the experts to roughly decide the level of agreement on each question.

Round 1 survey consisted of 59 evaluation questions categorized in 11 domains. It was distributed via personal emails. Experts were asked to rank their level of agreement with each statement on the 5-Point Likert Scale. There was an option for the experts to provide written comments for each question, suggest modifications, and/or offer justification for their ranking scores. If comments were provided, keywords and ideas were extracted. The comments were critically evaluated to determine if and what revisions were indicated. Not all respondents provided comments to support their scoring decision. According to the experts’ comments, seven domains did not reach a consensus. Therefore round 2 surveys consisted of 36 questions categorized in 7 domains. Finally, 56 evaluation questions were included in the FGD.

The authors analyzed the responses and extracted the recommendations from the participants’ responses. Then they devised a list of adaptations, which were approved subsequently by all the authors. A second set of evaluation questions were generated based on a second consensus meeting done by the researchers (SA, AK, NN).

### Step 3: virtual focus group discussions

Two virtual FGDs were conducted with medical educators who were formally invited based on convenience non-probability sampling method.

### First virtual FGD

A total of 30 members participated. They varied in gender, specialty, academic rank, and affiliation. Precautions were taken to guarantee both the anonymity of the participants and the confidentiality of their contributions to the discussions (e.g., their identities were concealed during data analysis).

Participants were divided in to five groups, with one of the authors moderating the session. The FGD lasted for 90-min, during which each moderator used a question guide aiming at exploring participants’ views on indicators for the already developed evaluation questions.

### Second virtual FGD

The methodology followed in second FGD was very much similar to the first FGD. However, the purpose of second FGD was to elicit the views of the participants regarding the data sources for the previously agreed upon indicators based on their personal experience in FDP, This was done in order to ascertain data relating to what is currently being used in the real practice.

The questions in the focus group guide covered five major themes concerning FDP based on the 5 × 2 D model: Decide (context and selection of trainees), Define (needs assessment and objectives), Design (materials and methods), Direct (communities of practice (CoP) and learning) and Dissect (key performance indicators (KPIs) and feedback).

The kickoff of the FGD was in the form of leading sentences and questions that are summarized in Textbox 1.

## Results

### Delphi results

The experts proposed a total of 42 modifications to the original 11 domains, ranging from 1 to 5 modifications per domain. Some of the modifications consisted of minor wording changes (i.e., “mechanism” instead of “structure” in domain G) while other suggestions were more extensive (i.e., merge / discard / add more details to enhance comprehension). Round 1 of the Delphi process began with 11 domains (59 questions). The 19 experts accepted 4 of the proposed domains, modified the remaining 7 domains. Overall, the experts directed most suggestions to domain B and G (9 modifications), with the fewest suggestions made to domain E (3 modifications). Some domains received no comments and reached consensus at round 1. Therefore, they were not included in Delphi round 2. The 2nd round included 7 domains (36 questions). Eighteen experts responded to our invitation and agreed to participate in round 2. All domains reached a consensus by the end of round 2 as shown in Table 1. In summary, the consensus in round 1 was 88.3% while all the questions reached 100% consensus by the end of round 2 (Table 1).

**Table 1:** Delphi Scores in Round 1 and 2

	Round 1 Delphi (***n*** = 19)			Round 2 Delphi (***n*** = 18)		
Questions	Number of experts agreed on the question	Mean	Percentage of consensus	Number of experts agreed on the question	Mean	Percentage of consensus
Domain A						
A1- Has the context of the training been well defined? ^a^	17	4.4	89.5	17	4.6	94.5
A2- Is it mentioned in the faculty development program description? ^b^	14	4	73.7	16	4.2	88.8
A3-Does the context identify the potential target audience? ^a^	17	4.3	89.5	16	4.6	89.8
A4-Does the context identify the specific need or situation necessitating the training? ^a^	17	4.3	89.4	17	4.7	94.5
A5-Does the context identify the physical attributes to the needed training? ^b^	15	4	79	16	4.4	88.9
A6-Is the program aligned with emerging trends in faculty development like blended learning, online learning, competency-based education.... etc.? ^a^	17	4.5	89.5	18	4.7	100
Domain B						
B1-Are the faculty selected for the program identified? ^a^	16	4.4	84.2	15	4.2	83.3
B2-Are the faculty selected for the program stratified according to their knowledge? ^a^	14	3.9	73.6	15	4.2	83.3
B3-Are the faculty selected for the program stratified according to interest? ^a^	14	3.9	73.7	14	4.1	77.7
B4-Are the faculty selected for the program homogenous in terms of knowledge and interest? ^b^	10	4	52.6	16	4.1	88.7
B5- Is there a degree of heterogeneity employed in the selection of the trainees? ^d^				16	4.1	88.7
Domain C						
C1-Have the trainee needs been studied? ^a^	17	4.5	89.5	17	4.5	94.5
C2-Have the identified needs been prioritized? ^b^	16	4.3	84.2	17	4.5	94.5
C3- Have the needs been reflected on the content or methods of training? ^b^	16	4.5	84.2	17	4.5	94.5
C4- Have the institutional needs been studied? ^b^	16	4.3	84.2	18	4.6	100
C5- Have the identified needs been prioritized? ^e^	16	4.3	84.3			
C6- Have the needs been reflected on the content or methods of training? ^e^	16	4.4	84.2			
Domain D						
D1-Are there defined objectives for the training? ^a^	16	4.5	84.2	18	4.8	100
D2-Are the objectives SMART? ^a^	16	4.3	84.2	18	4.8	100
D3-Are the objectives aligned with any of the identified needs? ^a^	15	4.3	79	18	4.8	100
D4- Are there objectives that deal with trainee soft skills? ^c^				15	4.4	83.3
Domain E						
E1-Are there materials for the training? ^a^	15	4.2	79	18	4.6	100
E2-Are the materials authentic? ^a^	15	4.1	78.9	17	4.5	94.4
E3-Are the materials in proper format? ^a^	15	4.1	79	18	4.5	100
E4-Are the materials adequate for the training content? ^a^	16	4.2	84.2	18	4.7	100
Domain F						
F1-Are the instruction methods planned? ^a^	16	4.3	84.2	18	4.6	100
F2-Are there proper guides for instruction? ^a^	17	4.5	89.5	18	4.7	100
F3-Are they suitable for the content/ objectives? ^c^	17	4.5	89.5	18	4.6	100
F4-Are they suitable for the trainees? ^c^	16	4.1	84.2	18	4.5	100
F5-Are they innovative? ^a^	14	3.9	73.6	16	4.6	88.9
F6-Are they feasible? ^c^	17	4.5	89.5	18	4.7	100
F7-Is the program longitudinal? ^a^	15	4	78.9	15	4.4	83.4
Domain G						
G1-Is there a proper structure to enable follow up of the learning? ^c^	15	3.9	78.9	16	4.4	88.9
G2-Is this structure adequate to the objectives? ^c^	14	4.1	73.6	17	4.4	94.4
G3-Is this structure known to everyone in the program (management, faculty, learners, administration)? ^c^	16	4.1	84.2	17	4.7	94.4
G4-Are there proper follow up tools for the learning? ^e^	14	3.9	73.7			
G5-Have the program ILOs been reached? ^a^	16	4.4	84.2	18	4.7	100
G6-Is there a method to assess the ILOs? ^a^	16	4.4	84.2	18	4.7	100
G7-Is there a methodology to deal with the non-attaining learners? ^b^	15	3.9	78.9	15	4.3	83.3
Domain H				Domains H, I, J & K were not included in Delphi round 2		
H1-Is there a platform to allow for building the community?	17	4.3	89.5			
H2-Is there time allocated in the program to allow for building the community?	16	4.1	84.2			
H3-Are there designated activities to allow for building the community?	16	4.3	84.2			
H4-Do trainees have enough knowledge of other trainees?	16	4.1	84.2			
H5-Are there collaborative efforts between trainees?	17	4.4	89.5			
H6-Are there enough collaborative project outcomes with trainees as project members (publications, conferences, workshops…etc.)	16	4	84.2			
Domain I						
I1- Has the program achieved growth over the years? (Number of attendees, learner satisfaction, learner attainment, measurable impact on teaching/ learning/ assessment…etc.)	16	4.4	84.2			
I2- Are there established methods to measure the KPIs?	15	4.2	79			
I3- Is there a dedicated team for measuring the KPIs?	15	4.3	79			
I4- Is there enough data collected?	15	4.2	78.9			
I5- Is the data properly analyzed?	15	4.1	79			
I6- Is the information deduced from the data properly reported/ discussed?	15	4.3	79			
I7-Are there corrective actions taken based on the information deduced?	15	4.2	79			
Domain J						
J1- Has the feedback improved over the years? (Student satisfaction/ faculty satisfaction/ student attainment)	15	4.2	78.9			
J2- Are there established methods to measure the learner and trainer feedback?	15	4.3	79			
J3- Is there a dedicated team for measuring the learner and trainer feedback?	16	4.4	84.2			
J4- Is there enough data collected?	15	4.1	78.9			
J5- Is the data properly analyzed?	16	4.3	84.2			
J6- Is the information deduced from the data properly reported/ discussed?	16	4.2	84.2			
J7- Are there corrective actions taken based on the information deduced?	16	4.3	84.2			
Domain K						
K1- Are there decisions and or practices signifying non-linear training plan methods? E.g. Revising content while directing the learning… etc.	15	4	79			

^a^Same in Round 1 and 2

^b^Reformulated after Delphi round 1

^c^Reformulated/wording

^d^Newly added in Delphi Round two

^e^Discard

### FGD results

The final version of the evaluation questions after Delphi round 2 (56 questions) were used for discussion and generation of the indicators and data sources as shown in (Table 2).

**Table 2:** Evaluation guide for faculty development program in educational effectiveness

Evaluation question	Indicators	Data Sources	Data Collection Method
**Domain A: Context**			
A1- Has the context of the training been well defined?	• Provide a description of the training context in printed and/or online format	• Program specification/ Faculty guide/ Brochures • Surveys/ Website	• Document review • Website Review • Survey Review
A2- Is the context described in the faculty development program description?	• Provide an orientation of the program context to the trainees	• Faculty guide/ Brochures/ Program specification/	
A3-Does the context identify the potential target audience?	• The context is specifically designed with the target audience in mind. • There is a description of the intended target audience in the program specifications. • Percentage of trainees that see that the program meets their needs.	• The FDP mission and vision and objective statements • Preamble of the course/ Program specs/ Brochures/ Faculty guide	
A4-Does the context identify the specific need or situation necessitating the training?	• There is a description of the specific need or situation in the program specifications	• Survey for a needs assessment.	
A5-Does the context identify the place and time?	• Description of the place and the program’s timeframe in the program specification	• Preamble of the course/ Program specs/ Brochures/ Faculty guide	
Domain B: Faculty			
B1-Are the faculty selected for the program identified?	• Presence of admission criteria with a clear description of the target audience	• Program specifications	• Document review • Surveys
B2-Are the faculty selected for the program stratified according to their knowledge?	• Presence of training program pre-requisite	• Faculty guide/ Brochures	
B3-Are the faculty selected for the program stratified according to interest?	• Survey the trainees and trainers’ interests upon admission/registration.	• Compare group allocation form with the registration forms	
B4- Is the selection of the trainees for the program homogenous in terms of knowledge and interest?	• Review attendance sheets (Registered Vs attended)	• Compare the attendance list and registration form	
B5- Is there a degree of heterogeneity employed in the selection of the trainees?	• Presence of training program pre-requisites indicating a wide range of variables (sex, race, country, specialty.)	• Program Specifications	
Domain C: Needs			
C1-Have the trainee needs been studied?	• Trainees’ knowledge gaps and training requirements were identified as per the literature review. • Percentage of trainees expressing willingness to attend FDP in the ‘identified topic.’ • Percentage of trainees mentioning this topic in their Personal development plan	• Relevant literature articles • Documentation of faculty needs assessment. • questionnaire • Faculty members personal development plans	• Document review • Review of media files • survey • FGD / Interview with trainees
C2- Have the institutional needs been studied?	• Quality assurance report suggests that this topic needs improvement. • A review of the literature reveals that institutions need to train their faculty in the “identified. • Leadership/administrators/curriculum committee / medical education/quality assurance believe that there is scope for improvement in the ‘identified domain’ and recommend FDP	• Quality / accreditation report • Documentation of relevant literature review/ Soft or hard copy of relevant journals • Documentation of institutional needs assessment questionnaire/ • Expressed oral and written opinions of Leadership/administrators/curriculum committee / medical education/quality assurance/ Documentation of ‘learner’ needs assessment questionnaire.	
C3-Have the identified trainee and their institutional needs been prioritized?	• Percentage of dissatisfaction from trainees regarding this identified topic/domain. • Percentage of trainees and administrators who believe that these tasks/contents/training in the ‘identified topic’ should be given high priority	• Documentation of ‘prioritization’ based on the Data sources of C1 and C2/ FDP schedule/ Brochure. • Trainee and administrators’ feedback/satisfaction questionnaire	
C4-Have the identified trainee and their institutional needs been reflected on the content and methods of training?	• Percentage of identified trainees and their institutional needs added as contents with appropriate tasks and methods for training in the FDP schedule. • The proportion of experts who agree that trainee and their institutional needs been reflected on the content and methods of training	• Teaching materials/handout/ Recording of FGD with experts/ • External reviewer report	
Domain D: Objectives			
D1-Are there defined objectives for the training?	• Expected outcomes/contents of the FDP are mentioned as well-defined objectives. • The proportion of experts who agree that objectives are well defined for the training.	• FDP schedule/ Brochure/ Reading materials / Handouts • Recording of FGD with experts/ External reviewer report.	Document review FGD FGD with experts (Comparison of FDP schedule, FDP with results of faculty needs assessment/literature review / institutional needs assessment)
D2-Are the objectives SMART?	• The proportion of experts who agree that objectives are specific, measurable, achievable, (or agreeable), realistic (or relevant) and time-bound, (or timely) • Percentage of program organizers who agree that the objectives were SMART.	• FDP schedule/ Brochure/ Reading materials / Handouts/ External reviewer report/ Recording of FGD with experts • Analysis of Feedback questionnaire.	
D3-Are the objectives aligned with any of the identified needs?	• Percentage of trainees/administrators who agree that identified objectives are aligned with either trainee or their institutional needs. • The proportion of experts who agree that trainee and their institutional needs been reflected on the content and methods of training	• Trainee and administrator questionnaire with analysis reports • FDP schedule/ Brochure/ Documentation of faculty needs assessment questionnaire with analysis/ Documentation of institutional needs assessment/ Recording of FGD with experts/ Inter-rater analysis of experts.	
D4- Are there objectives that deal with trainee soft skills?	• Percentage of identified objectives that are dedicated to soft skills of the trainees (Under regular circumstances) • Percentage of adapted objectives that deal with trainee soft skills (Under special circumstances)	• Analysis of survey from trainees / resource faculty • /administration/ FDP schedule with contents/ Teaching materials / handout / Analysis of Expert opinion	
Domain E: Materials			
E1-Are there materials for the training?	• There is the availability of pre-reading materials, timetables and schedules are provided.	• FDP content/ Lesson outlines/ Brochures/ Timetables	• Interview • Document review • Survey
E2-Are the materials authentic?	• Materials are tailored to the institution’s and trainees’ demands. • Materials are suitable to the context of the institute, culture, and country.	• Literature review (on authentic resource material)/ Guidelines of the institute/ Need’s assessment report • FDP program content	
E3-Are the materials in proper format?	• The program is well structured with proper learning objectives and timelines.	• Lesson outlines/ Study guides/ Trainee interviews/ Guideline from literature review/accreditation bodies	
E4-Are the materials adequate for the training content?	• Materials are found sufficient to cover the domain of FDP e.g., Teaching and learning /Leadership/ Workplace-based assessment etc.	• FGD of facilitators • External reviewer report/ End of program trainee survey/ End of program trainer survey	
Domain F: Methods			
F1-Are the instruction methods planned?	• Instruction methods are well described.	• Lesson plans/ Questionnaires to the trainees/ Peer observation/ Trainee interviews • FDP program syllabus	• Documents Review • Survey • Observation • Digital data review • Interviews • Document review
F2-Are there proper guides for instruction?	• There is a document guiding students about the outline of the instruction.	• Lesson outlines/ Study guides	
F3-Are the instruction methods suitable for the content and objectives?	• There is a variety of instruction material that delivers the content most efficiently in the opinion of experts, trainers and trainees	• Lesson outlines/ Study guides	
F4-Are the instruction methods suitable for the trainees?	• Percentage of trainees who pass the attainment level of the program. • More than 70% of the trainees are satisfied with the instruction methods	• Student assessment result/assignment results • Collection of expectations of the trainee at beginning of the session and matching with the objectives detailed throughout the session • (Surveys /Discussions Teaching Learning Conversation). • A study with constructive alignment in planned, delivered and assessed material. • Student end of program reports • Student satisfaction surveys.	
F5-Are there innovative instruction methods in the program?	• Innovative methods such as different approaches like gamification, TBL, role play, Case-based learning etc. are present.	• FDP brochure/ promotion from the institute / Software used. • Interview the participants. • Comparison study of innovation and previous program methodology • Brainstorming and group discussion	
F6-Are the instruction methods feasible?	• Instruction methods are found feasible by external reviewers. • Percentage of instruction methods reported that are performed	• Report of external reviewers/ • Trainee and trainer feedback	
F7-Is the program longitudinal?	• The program runs longitudinally for more than 3 months with an opportunity for self-study and structured assignments.	• Syllabus/ Faculty guides	
Domain G: Learning oversight			
G1- Is there a functional process to enable follow up of the learning?	• Percentage of the trainees passing the formative assessment. • Two-three formative assessment exams are conducted each module. • Improvement of the student performance • Trainee reflections are collected at fixed intervals	• Records of the training sessions • Reflection reports • Mentor report and self-assessment report • Pre and post-test results	• Document review • Surveys • Observation • Statistical analysis • Assessor evaluation checklist • Questionnaire • Focus group • website review
G2-Is this mechanism adequate to the objectives?	• Trainees’ perception of the concepts indicates that the mechanism is adequate. • Percentage of the non- attaining Trainees diagnosed annually. • Percentage of the procedural defects detected by this mechanism.	• Trainees’ feedback • Audit report	
G3-Is this mechanism known to everyone in the program (management, faculty, learners, administration)?	• Percentage of trainees and administrators who received the announcement and program details. • Percentage of the student accessing the website and knowing the mechanism. • Percentage of students’ satisfaction with the mechanism. • Use of all the available communication channels emails, brochures, social media platforms.	• Emails and brochures • Website metrics • Questionnaire results • Emails, brochures, social media groups	
G4-Are there functional measurement tools to evaluate the learning and skill acquisition?	• There are differentiating assessment tools to assess learning	• Ensure the validity and reliability (Psychometrics measures) through: • Multiple tools • Multiple occasions • Multiple assessors (external assessors)	
G5-Have the program ILOs been reached?	• The student success rate in assessments and post-tests • Student satisfaction feedback questionnaires and percentage of students agreeing that the ILOs have been achieved.	• Post evaluation quiz Statistical analysis report • Questionnaire results	
G6-Is there a method to assess the ILOs?	• There is a program post-test or program evaluation that demonstrates learners’ achievement	• Post-test results • Program Evaluation report	
G7-Is there a methodology to deal with the non-attaining learners?	• Percentage of the non-attaining learners that have undergone a remedial procedure. • Percentage of the trainee informed and aware of the remedial policy • An Authorized policy is announced to the trainee • Frequency of evaluation measures to detect the non- attaining learners	• Mark list • Learner feedback • PDF brochure/ website • Evaluation reports	
Domain H: Community of practice			
H1-Is there a platform to allow for building the community?	• There is a platform that is user friendly, flexible and allows for communication between trainees.	• Platform dashboard • Trainee and trainer feedback	• Observation • Surveys • Document review • Digital review
H2-Is there time allocated in the program to allow for building the community?	• Percentage of time allocated for activities established to promote community building	• Program specifications/ schedules	
H3-Are there designated activities to allow for building the community?	• Presence of activity moderators/ Facilitator to help them build the community	• Program report	
H4-Do trainees have enough knowledge of other trainees?	• Activities allocated for community building are innovative. • Availability of trainee information on platforms and/or in printed format	• Website	
H5-Are there collaborative efforts between trainees?	• Percentage of trainees that built a relationship with other trainees (Projects, publications, social media friendship or social activities).	• Survey • Publications • Social media • Project proposals	
H6-Are there enough collaborative project outcomes with trainees as project members (publications, conferences, workshops…etc.)	• The number of collaborative projects established between members in each group. • The number of joint activities between trainees yearly (conferences, publications etc.) • Impact evaluation of joint activities	• Surveys • Annual alumni reports • Impact evaluation report	
Domain I: KPI			
I1- Has the program achieved growth over the years? (Number of attendees, learner satisfaction, learner attainment, measurable impact on teaching/ learning/ assessment…etc.)	o **Number of attendees** • An annual increase in the number of trainees attending the program • An annual increase in the number of trainees applying to attend the program • Percentage of the increase in the number of trained trainees compared to non-trained faculty members annually • Trainee satisfaction • Average of trainees’ satisfaction rate with the activities of the training program on a five-point scale in the program evaluation survey • Trainee attainment • Increase in the proportion of trainees who • complete the program in minimum time. • Increase in the proportion of trainees passing the program annually • Improvement in scores of the trainees in the post-program assessment than pre-program assessment • Dropout rate/ total program • Number of complaints/ year • Recommendation of the program • Measurable impact on teaching, learning and assessment • Percentage of trainees who graduated from the program who were appointed in leadership positions • Percentage of graduates promoted • Improvement of skill the of graduates in the workplace	• An official document with the number of trainees entering the program annually • An official document with the number of trainees graduating from the program for one batch • FG recordings • Feedback from colleagues and students	• Observation • Self-assessment questionnaires • Trainee survey • FGD / Interview with trainees • Document review • Statistical data analysis
I2- Are there established methods to measure the KPIs?	• Valid and reliable established methods for measuring KPI • Timely and continuous measuring of the KPI.	• Evaluation Reports • Annual reports • Data collection tools	
I3- Is there a dedicated team for measuring the KPIs?	• A dedicated and professional team for measuring each of the KPI is appointed	• Appointment decree for the team	
I4- Is there enough data collected?	• Adequate data collection for measuring each of the KPIs	• Documents • Records • Statistical data	
I5- Is the data properly analyzed?	• Proper analysis of the data using suitable statistical methods for all KPIs	• Documents • Records • Statistical data	
I6- Is the information deduced from the data properly reported/ discussed?	• 80% of the information deduced from the data properly reported/ discussed • Increase in the number of Scientific council meetings that discuss the deducted information properly	• Meeting minutes of the scientific councils	
I7-Are there corrective actions taken based on the information deduced?	• Presence of proof of corrective action taken in response to assessment results. This can be a change in the scope, structure or content of the program.	• Program report	
Domain J: Feedback			
J1- Has the feedback improved over the years? (Student satisfaction/ faculty satisfaction/ student attainment)	• **Trainee satisfaction** • An annual increase in the satisfaction rate of trainees, faculty and administration of 10% • **Trainee attainments** • Percentage of the trainee who passed the course improved by 10%	• Surveys • FG and interviews • Post-training quizzes	• Focus groups • Interviews • Statistical analysis • Document review • Observation
J2- Are there established methods to measure the learner and trainer feedback?	• There are valid and reliable established methods for measuring feedback (end of program surveys, focus groups, reflection meetings) • Timely and continuous measuring of the feedback	• Report from external program reviewers • Data sets available from the feedback	
J3- Is there a dedicated team for measuring the learner and trainer feedback?	• A dedicated and professional team for measuring each of the feedback	• Appointment decree for the team	
J4- Is there enough data collected?	• There exists at least one type of data set for each KPI	• Data repositories for the program	
J5- Is the data properly analyzed?	• Data is analyzed using a well-established data analysis program	• Programs existing on the computers where data repositories are present • Data repository formats	
J6- Is the information deducted from the data properly reported/ discussed?	• The information deducted from the data properly reported/ discussed in the relevant scientific committees	• Minutes of meeting of relevant scientific committees	
J7- Are there corrective actions taken based on the information deduced?	• At least one annual corrective action can be demonstrated	• Program report • Program specification of the upcoming training round	

## Discussion

The main focus of this work was to develop a guide for evaluating longitudinal faculty development programs. In order to do that, expert opinions were taken into account. The reliance on expert consensus was previously used by Minas and Jorm and Kern [23, 24].

Recent trends in training of proficient educators in HPE for their newer roles and responsibilities demand a shift to longitudinal FDPs (LFDPs) [14, 25, 26]. LFDPs developed based on robust models are shown to steadily establish and strengthen the desired competencies of the participants [27].

Even though several linear models were proposed in the past [11–13, 28–33], there was an explicit need for a flexible cyclical model that is more appropriate for LFDPs [9, 20, 34].

To achieve this objective, multi-level analysis, a widely used scientific method was employed [35–37]. This qualitative method was built upon the input from individuals with vast experience in planning and implementation of FDPs, engrained on a series of trials and errors encountered in the past [23, 24].

### Community of Practice (CoP)

In this study, there is an inclination to identify indicators to test the continuity of the community practice. There is a multitude of facets used starting from the availability of information to the methods and platforms for communication to the impact of product development because of ongoing collaborations. The use of similar indicators to evaluate the development and sustainability of CoP was described before in previous work [38, 39].

Evaluating the CoP practice requires a longitudinal approach that allows for visiting and revisiting preset indicators [40]. This requires a communication strategy with alumni communities and a methodology to keep them engaged throughout the testing period.

CoP develop over five stages according to Etienne and Beverly Wenger-Trayner, 2015 [41].

Each of these stages requires an evaluation strategy and a set of indicators to identify the success of the process [38, 39]. In this study, indicators are stratified across all the five stages of CoP.

### Data collection methods

In this study there are three sets of data collection methods for evaluation; 1) observation, 2) interviews, surveys or focus groups and finally 3) document or media review. According to Peersman, G. (2014), data collection tools are either those collected by direct observations, those reported by stakeholders either through interviews, surveys or focus groups and those extracted from evidence which might be documents or media analysis. This is in concordance with our proposed data sources [42].

### Selection of faculty

Selection of the faculty for the training program received a semi-consensus with a tendency to identify indicators to test the homogeneity in terms of knowledge and interest among the faculty recruited for the program. Effective training design reduces the evaluation and categorization effort for the participants by building on pre-existing sector knowledge and expertise [43]. Therefore, many programs have a few salient requirements which will need to be met by the faculty to join the advocacy program services.

In terms of training alliance, focusing on the faculty selection with homogenous knowledge and interest will decrease the knowledge power gaps between the participants focusing on a common goal to improve and develop. Believing that candidates should possess several relevant qualities, the literature did not shed the light on the indicators required for that. This was attributed by some authors to the fact that faculty development is embedded within the training system with a systematic dynamic trainee evaluation [44, 45].

However, heterogeneous groups can outperform homogeneous groups in terms of the range of decision options and consequences of decisions that they consider [46, 47]. Thu s, a degree of heterogeneity is allowed depending on the goal and outcomes of the training program.

### Quantification

When experts were requested to contemplate the standards, it became evident that quantification was a prerequisite for agreeing upon setting benchmarks. Similar views were resonated by other researchers as well [48–52]. Recognition of this fact strengthens the need for regional standards that fit seamlessly to cater to the requirements of institutions in diverse areas. Thus, the identified set of standards and indicators are meant as a guide for LFDPs with due adaptations to suit local needs [53, 54].

### Limitations of the study

This work did not cover aspects of validation of the tool that can be performed longitudinally over a period of time. This work could benefit from a further study and application of this evaluation guide in real life situations, and this can be a future direction of research. Next steps recommended will be to implement the evaluation model on a pilot basis taking into account utility in various contexts. A study is also recommended to compare the novel model with existing models like Kirkpatrick model regarding process and outcome.

## Conclusion

Conducting faculty development is an art that needs a degree of flexibility within the scope of ensuring a continual process of improvement and ongoing learning. The use of the guide for best practice in faculty development can be a self-evaluation tool as well as a quality assurance tool for external auditors. The best practice guide together with the evaluation process is a universal technique that can be adopted worldwide where indicators can be quantified based on local context after it has been tested for applicability, usability, and utility.

### Recommendations

This work offers direction for schools needing to perform and evaluate FDPs. Using the checklist in Table 2 can be a good guide for schools in the evaluation and continuous quality assurance cycle. It is recommended to incorporate a structured strategy for evaluation, as early as possible while planning for FDPs.

## Supplementary Information

**Additional file 1.**

## Data Availability

The materials are video recordings and surveys. Data set are available at Harvard Dataverse. The data and materials can be accessed at DOI: 10.7910/DVN/NNRS0R, Harvard Dataverse, V1.
